# Oxidative Degradation of Hazardous Benzene Derivatives by Ferrate(VI): Effect of Initial pH, Molar Ratio and Temperature

**DOI:** 10.3390/toxics9120327

**Published:** 2021-12-01

**Authors:** Dian Majid, Il-Kyu Kim, Fajar Budi Laksono, Aditya Rio Prabowo

**Affiliations:** 1 Department of Environmental Engineering, Universitas PGRI Adi Buana Surabaya, Surabaya 60234, Indonesia; majid@unipasby.ac.id; 2 Department of Environmental Engineering, Pukyong National University, Busan 48513, Korea; ikkim@pknu.ac.kr; 3 DTECH Engineering, Salatiga 50742, Indonesia; fajar@dtech-engineering.com; 4 Department of Mechanical Engineering, Universitas Sebelas Maret, Surakarta 57126, Indonesia

**Keywords:** bromobenzene, chlorobenzene, ferrate, oxidation

## Abstract

Two of the most hazardous benzene derivatives (HBD) that have polluted the aquatic environment are bromobenzene and chlorobenzene. Ferrate can degrade various pollutants quickly and efficiently without producing harmful byproducts. This study aims to determine the ability of ferrate to degrade harmful contaminants such as bromobenzene and chlorobenzene. A series of batch experiments were carried out, including for the molar ratio, initial pH solution, and temperature. The study was conducted at an initial pH of 3.6 to 9.6, a molar ratio of 2 to 8 and a temperature of 15 to 55 °C. The study will also examine the differences in functional groups in these pollutants. As a result of the experiments, the optimum conditions to oxidize HBD in a batch reactor was found to have an initial pH of 7.0, a molar ratio of 8, and a temperature of 45 °C, with a 10 min reaction time. Ferrate has a degradation ability against chlorobenzene greater than bromobenzene. The functional cluster in pollutants also significantly affects the degradation ability of ferrate. The results of the degradation experiment showed that ferrate(VI) could effectively oxidize hazardous benzene derivatives in a solution.

## 1. Introduction

Hazardous benzene derivative (HBD) compounds are of great concern among the group of major environmental pollutants because they have toxicological and ecological implications. Two HBDs that have polluted the aquatic environment are bromobenzene and chlorobenzene [1,2]. Bromobenzene and chlorobenzene have broad uses in industrial applications, agriculture, colorant substances, solvents, and much more [3,4]. Alongside their range of uses and essential roles in the industrial field, the level of use and the level of HBD contamination are increasing. On the other hand, bromobenzene and chlorobenzene have adverse effects on the environment due to their naturally low biodegradation process, known to have toxicity [5,6], which is dangerous for the natural aquatic environment biota. Therefore, there is a need for a proper method in overcoming the pollutant effects of bromobenzene and chlorobenzene waste before being released into the aquatic environment.

Certain methods have been developed by previous research in addressing various types of similar pollutants. These methods include absorption, bio-adsorption, photocatalytics, and ozones [7,8,9,10]. However, this method has not been effective and efficient in overcoming bromobenzene and chlorobenzene contamination due to its lengthy and complicated process. Therefore, alternative methods are needed to remediate bromobenzene and chlorobenzene contaminants in water.

A few decades ago, Iron (Fe) was found at an oxidation state of +6, called ferrate(VI), Fe(VI), which has the potential to oxidize in an environmentally friendly manner in water and wastewater treatment [11,12]. It has excellent water and wastewater treatment potential because Ferrate(VI) has a reduction-oxidation potential of ferrate ions (VI) as high as 2.2 V in acidic and 0.72 V in alkaline conditions. Ferrate has a unique ability as an oxidant at the beginning of the reaction, and then becomes coagulant because ferrate turns into Fe(III), which will increase the purification process [13].

Many researchers have studied ferrate’s ability to degrade organic and inorganic pollutants and colorant substances [14,15,16,17]. Its ability to degrade various pollutants makes ferrate(VI) a very promising method in water and wastewater treatment.

In this article, bromobenzene and chlorobenzene were used as models of HBD, and we thoroughly investigated the ferrate(VI) process for bromobenzene and chlorobenzene degradation. This study examines the performance of ferrate in the degradation of bromobenzene and chlorobenzene. Testing is performed on the initial pH solution and the molar and temperature ratio. The different effects of the functional groups of the two pollutants will also be studied.

## 2. Materials and Methods

### 2.1. Material

Ferric oxide (Fe_2_O_3_) and sodium peroxide (Na_2_O_2_) as primary materials were all obtained from Alfa Aeser (Tewksbury, MA, USA). Bromobenzene and chlorobenzene, also known as halogenated benzene (HB), were used to model the contaminant. Bromobenzene and chlorobenzene were bought from Sigma-Aldrich (St. Louis, MO, USA). Buffer solutions for pH adjustment (pH 4, 7 and 10) were all purchased from Samchun Chemicals Company (Seoul, Korea). 

### 2.2. Synthesis of Fe(VI)

Ferrate(VI) was prepared according to previous study methods with several modifications steps [18,19,20]. Fe(VI) synthesis used Fe_2_O_3_ as the main component. A total of 0.48 g Fe_2_O_3_ and 1.18 g Na_2_O_2_ were mixed and then heated at a temperature of 550 °C. The resulting mixture was oxidized using 30 mL NaOCl under alkaline conditions with 10 g NaOH. We mixed these ingredients with rapid stirring until a thick reddish-purple solution was formed. The synthesized Fe(VI) was filtered twice to remove impurities, then Fe(VI) was stored in a closed glass tube. Finally, ferrate was analyzed using UV-Vis spectrophotometry.

### 2.3. Experimental Procedures

**Figure 1:**
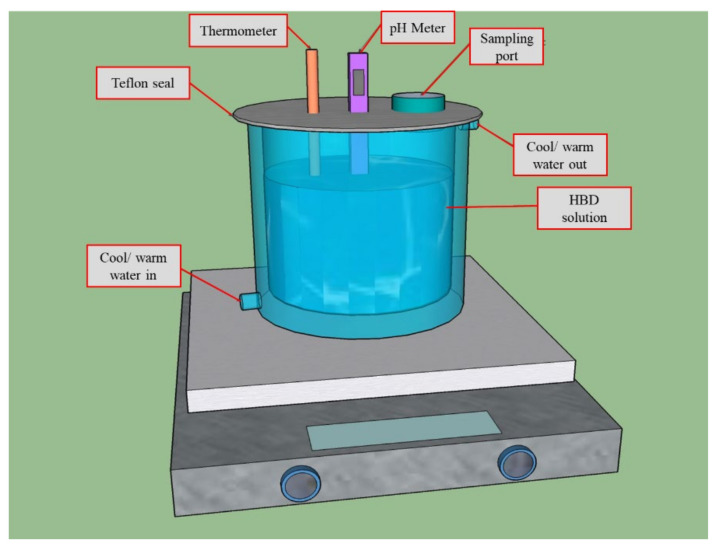
Batch reactor.

All the batch experiments for HBD degradation using ferrate (Na_2_FeO_4_) were performed (Figure 1). HBD solutions were prepared in distilled water. HBD degradation removal by Fe(VI) was investigated for the effect of pH (3.6 to 9.4), molar ratio (2 to 8), and temperature (15 °C to 55 °C). The initial pH was adjusted using a fabricated buffer solution, NaOH and sulfuric acid. The temperature was controlled using a temperature control bath. The degradation experiment was started by injecting a specific Fe(VI) dose into the reactor with stirring. At a specific time (0.5, 1, 2, 5, 10 and 20 min), a sample was taken using a water sampler, and then the sample was extracted using the liquid–liquid extraction technique with hexane. 

### 2.4. Analytical Methods

In this study, the concentration of HBD was determined using GC-ECD (gas chromatography-electron capture detector) (Technologies Co. 4890D, Rx-5 ms column (L = 30 m, internal diameter = 0.25 m)) after going through the liquid–liquid extraction process. The initial temperature was 50 °C, the final temperature was 280 °C, the inject temperature was 280 °C, detect temperature was 300 °C, the rate was 50 °C/min, initial time was 2 min and final time 9 min, with a column length of 30 m and split-less mode. The HBD concentration measurement results will be used to calculate the % degradation efficiency using the following equation (Equation (1)), where $C_{i}$ is initial concentration and $C_{f}$ is final concentration:

(1) $$\mathrm{Degradation}\;\mathrm{Efficiency}\;\left(\%\right)=\frac{C_{i}-C_{f}}{C_{i}}\times 100$$

## 3. Results and Discussion

### 3.1. Synthesis of Fe(VI)

**Figure 2:**
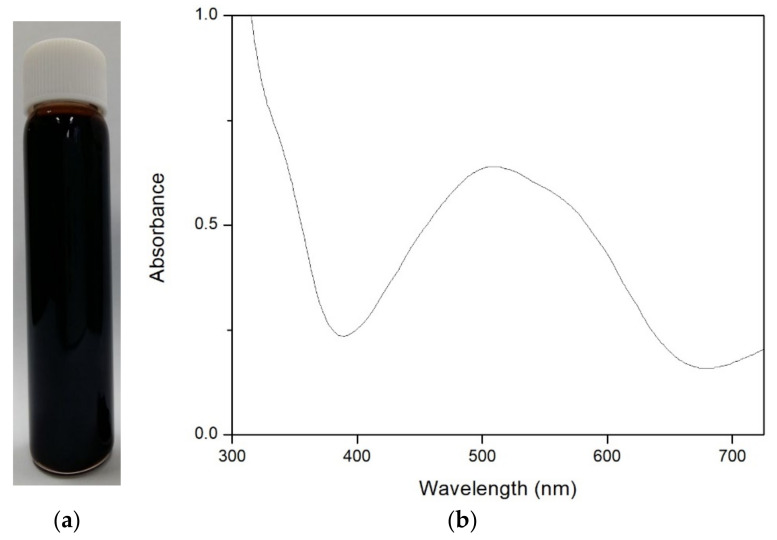
(**a**) Fe(VI) (Na_2_FeO_4_); (**b**) Adsorption spectrum (UV-Vis) of Fe(VI).

Fe(VI), FeO_4_^2−^ can be synthesized by various techniques [21]. This study used the wet oxidation technique. Additionally, Fe exists with alkaline earth groups such as Li, Na, K, Rb, Cs and Ag [22]. This study used Na as the alkaline earth, with the formula Fe(VI) to Na_2_FeO_4_ (Figure 2a). Ferrate in Na_2_FeO_4_ had to be synthesized in each experiment due to its low stability.

One method that can be used to analyze ferrate is using UV-Vis spectrophotometry. Fe(VI) has a typical UV-Vis absorption at a wavelength of 510 nm [23], as shown in Figure 2b. The following reaction can describe the formation of Fe(VI) [24] Equations (2) and (3):

(2) Fe2O3+Na2O2 →2NaFeO2+12O2

(3) $$2NaFeO_{2}+3NaOCl+2NaOH\;\to 2Na_{2}FeO_{4}+3NaCl+H_{2}O$$

### 3.2. Effect of Initial pH on Degradation of HBD

**Figure 3:**
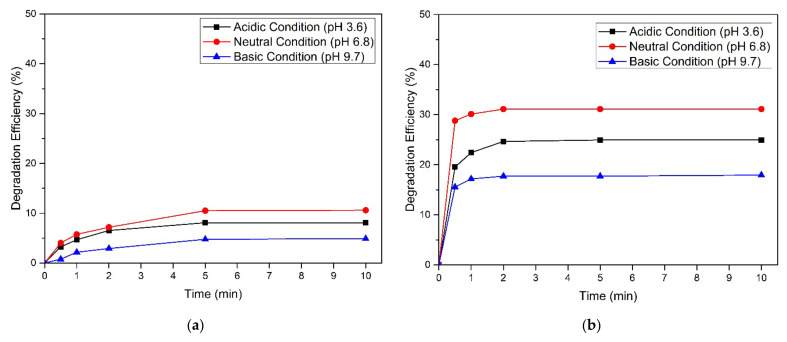
Effect of initial pH on the removal of (**a**) bromobenzene and (**b**) chlorobenzene. (Experimental conditions: C_o_ = 0.010 mM, [FeO_4_^2−^] = 0.010 mM, temperature = 25 °C).

The pH value is an important parameter affecting the ferrate process [25]. Varied initial pH solutions of HBD were tested from pH 3.6 to 9.7. The experiment was started by adding a dose of ferrate (Na_2_FeO_4_) to the reactor, i.e., 0.010 mM. The effect of pH on HBD degradation experiments by ferrate(VI) is shown in Figure 3. The oxidation of bromobenzene and chlorobenzene by ferrate occurs rapidly in the early minutes of the investigation, then rises slowly over time. A similar trend was obtained for the degradation of methylbenzene in a previous study [18].

According to Figure 3, quantitatively, bromobenzene and chlorobenzene removal at a low pH (3.6) was 8.09% and 24.961%. When the initial pH solution increased from 3.6 to 6.8, the levels increased obviously from 8.09% to 10.60% and 24.961% to 31.124%, respectively. Further, an initial pH solution increase to 9.4 reduced bromobenzene and chlorobenzene removal efficiency by 4.89% and 17.950%, respectively, in 10 min of contact time. According to previous research, at a low pH (<5), ferrate is very unstable and decreases rapidly (within minutes). The decomposition rate of ferrate with water is far greater than the reaction rate of ferrates with pollutants [26], whereas at a high pH (>9), ferrate is more chemically stable and lasts longer in the experimental reactor, resulting in the reaction rate of the ferrates tending to be slow [27]. Consequently, the experimental results showed that the highest bromobenzene and chlorobenzene degradation was achieved in neutral conditions (pH 6.8).

**Figure 4:**
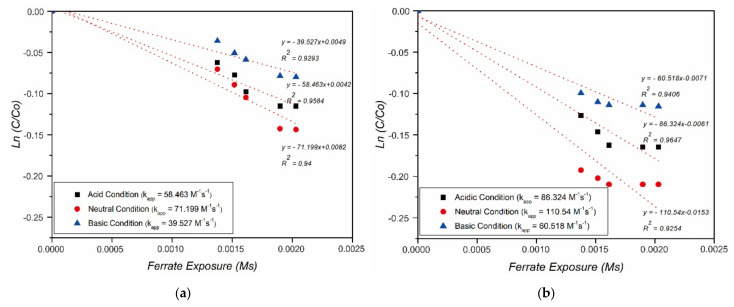
Rate of (**a**) bromobenzene and (**b**) chlorobenzene removal by ferrate(VI) at various pH levels.

Kinetic studies in the degradation of HBD by ferrate at various pHs were also carried out. The kinetics for the ferrate(VI) reaction and other, different compounds have been investigated and are very well determined by the law of second-order reaction rates [28,29,30,31,32]. Second-order reaction rate law following (Equation (4)) is rearranged and $d\frac{\left[HBD\right]}{dt}$ is integrated to become (Equation (5)):

(4) $$-d\frac{\left[HBD\right]}{dt}=k_{app}{\left[Fe\left(VI\right)\right]}_{\left(tot\right)}{\left[HBD\right]}_{\left(tot\right)}$$

(5) $$ln\frac{\left[HBD\right]t}{{\left[HBD\right]}_{0}}=-k_{app}\int_{0}^{t}\left[Fe\left(VI\right)\right]dt$$

where $ln\frac{\left[HBD\right]}{{\left[HBD\right]}_{0}}$ versus $\int_{0}^{t}\left[Fe\left(VI\right)\right]dt$, $\left[HBD\right]t$ and ${\left[HBD\right]}_{0}$ are HBD concentrations at time *t* and 0 s. The value of *k_app_* (*R^2^* = 0.9~) for the reaction of ferrate(VI) with bromobenzene and chlorobenzene degradation by ferrate(VI) oxidation in this study is shown in Figure 4.

The value of the rate constant (k) increased from 58.46 M^−1^s^−1^ to 71.19 M^−1^s^−1^ as the pH increased from 3.6 to 6.8, then decreased rapidly to 39.19 M^−1^s^−1^ for bromobenzene removal by ferrate(VI). The value of k increased from 86.324 M^−1^s^−1^ to 110.540 M^−1^s^−1^ as the pH increased from 3.6 to 6.8, then decreased rapidly to 60.518 M^−1^s^−1^ as the pH increased to 9.7 for chlorobenzene removal by ferrate(VI). It was shown that the highest k for HBD removal was achieved at neutral pH conditions. Additionally, chlorobenzene has a higher *k_app_* than bromobenzene removal.

### 3.3. Effect of Molar Ratio on Degradation of HBD

**Figure 5:**
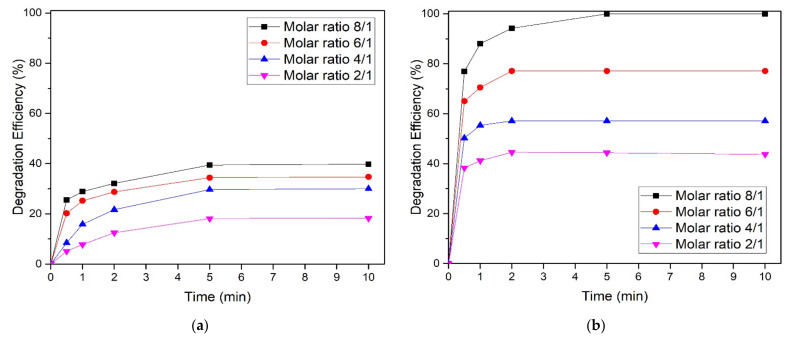
Effect of molar ratio on the removal of (**a**) bromobenzene and (**b**) chlorobenzene. (Experimental conditions: initial pH 6.8, C_o_ = 0.010 mM and temperature = 25 °C).

Increasing the molar ratio for the ferrate(VI) process generally enhances the degradation of contaminants in the solution [33]. A set of molar ratios ($\left[Fe\left(VI\right)\right]$/$\left[HBD\right]$) was freshly prepared from 2/1 to 8/1. Additionally, the fixed HBD (bromobenzene and chlorobenzene) solution was crafted at a concentration of 0.010 mM. The effect of the molar ratio on HBD degradation experiments by ferrate(VI) is shown in Figure 5. 

**Figure 6:**
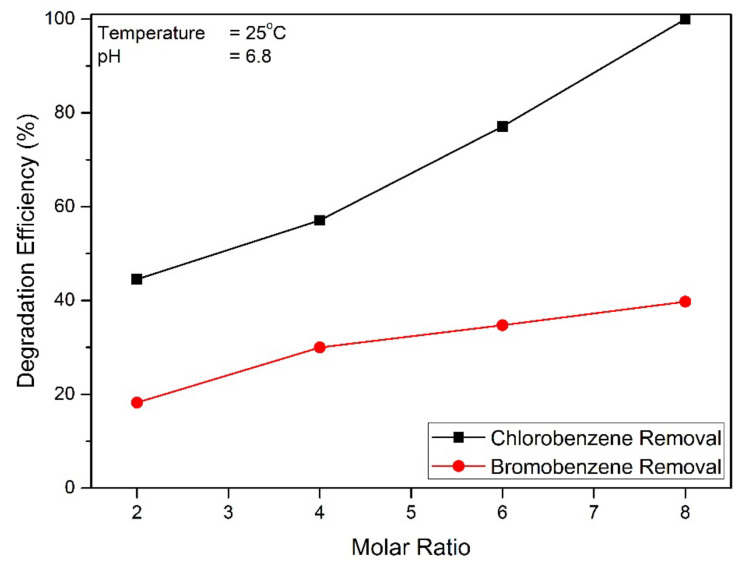
Relationship between molar ratio and degradation efficiency.

Increasing the molar ratio will significantly affect the degradation efficiency [34]. As shown in Figure 6, increasing the molar ratio from 2 to 8, the corresponding percent degradation efficiency decreases from 18 to 39% for bromobenzene degradation and increases from 44.53 to 100% for chlorobenzene degradation within a 10 min reaction time. The highest removal fraction of bromobenzene and chlorobenzene was approximately 44.530% and 100%, respectively, showing that ferrate effectively removed bromobenzene and chlorobenzene from water. It is interesting to note in this experiment that the pollutant degradation efficiency by ferrate increased with increasing molar ratio. Several reports for other pollutants indicate similar findings [13,35]. High degradation efficiency due to ferrate by-products can lead to the formation of Fe(III), which has abilities as a natural coagulant against pollutants. [36,37]. 

Complete degradation occurs in the degradation of chlorobenzene. Chlorobenzene degradation has a degradation efficiency almost two times greater than bromobenzene degradation. The electronegativity of pollutant functional groups (halogen: -Br and -Cl) also plays a very important role in the effectiveness of degradation. The electronegativity levels of the carbon–halogen bonds follow the following order: F-C > Cl-C > Br-C > I-C [38]. The level of electronegativity of pollutants will result in easier and better oxidation [39]. 

Previous researchers have proposed that the contaminant (X) degradation mechanism by Fe(VI) follows the following reaction mechanism (Equations (6)–(8)) [40]. On the other hand, the further decomposition of HBD by ferrate produces end products such as carbon dioxide [11,12].

(6) $$Fe\left(VI\right)+X\;\to Fe\left(IV\right)+X_{2}$$

(7) $$Fe\left(VI\right)+X\;\to Fe\left(IV\right)/Fe\left(V\right)+\mathrm{product}\;X$$

(8) $$Fe\left(VI\right)+X\;\to Fe\left(IV\right)+XO$$

For further studies of ferrate(VI) oxidation in this experiment, ferrate solutions with a molar ratio higher than 8 to 1 (ferrate(VI) to bromobenzene) were tested and analyzed to improve the bromobenzene degradation efficiency.

### 3.4. Effect of Temperature on Degradation of HBD

**Figure 7:**
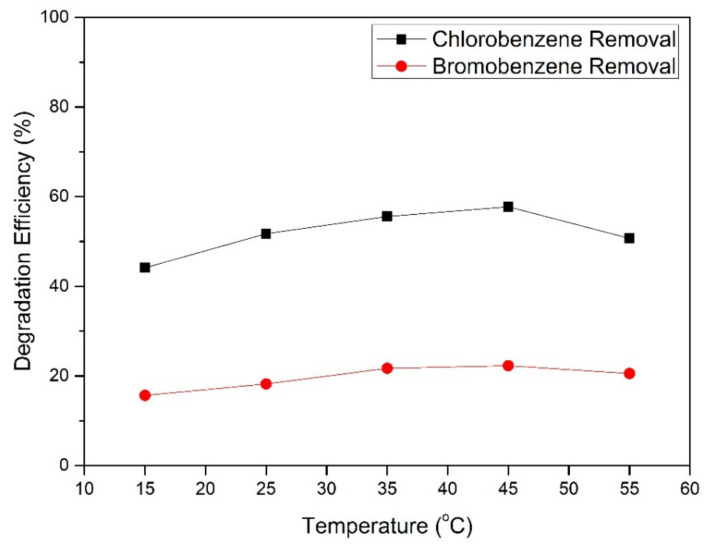
Effect of temperature on the removal of HBD by ferrate(VI). (Experimental conditions: C_o_ = 0.010 mM, [FeO_4_^2−^] = 0.020 mM, initial pH = 6.8).

Temperature is one of the critical parameters in the process of pollutant degradation using ferrate [27]. Temperature is a vital factor because it affects the stability of the ferrate performance; therefore, experiments were carried out at five different temperatures in degrading HBD. The influence of temperature on the reactivity of Fe(VI) with HBD degradation is shown in Figure 7. The degradation percentage of bromobenzene increased from 17.23 to 22.3%, and the degradation percentage of chlorobenzene increased significantly from 44.137 to 57.75% as temperature increased from 15 to 45 °C. It then decreased at temperatures above 45 °C, with a degradation efficiency of 20.12% for bromobenzene and 44.777% for chlorobenzene degradation. The optimal temperature condition in this study for the removal of HBD was observed at 45 °C. The experimental results make sense as reactants can only react when they are in contact, and molecules move faster and collide more frequently at higher temperatures (45 °C). However, at temperatures higher than 45 °C (for example, 55 °C), ferrates tend to be unstable. Other researchers have also confirmed these phenomena that the ferrate concentration decreases with an increase in temperature above 50 °C [41].

**Figure 8:**
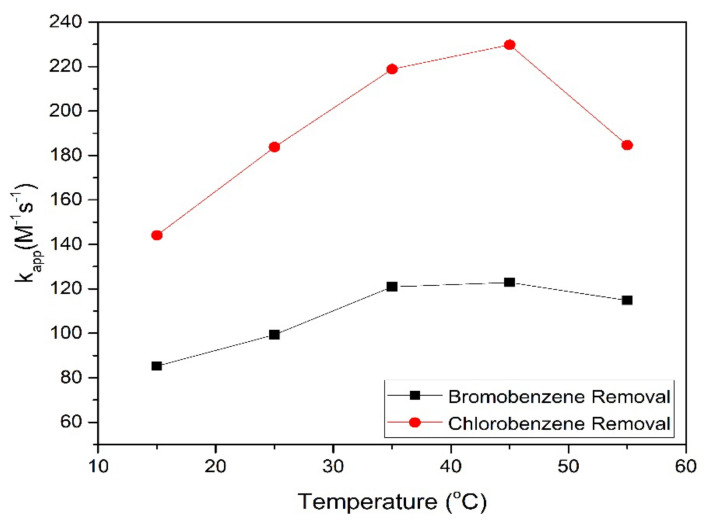
Relationship between T and *k_app_* value on HBD removal.

In general, temperature is very influential in the aqueous decomposition rate of ferrate [42]. As shown in Figure 8, the highest *k_app_* values were observed at 45 °C. The Arrhenius equation (Equation (9)) is used to determine the activation energy, and the activation energy value was found to be 81.6 KJ/mol for bromobenzene degradation and 80.540 KJ/mol for chlorobenzene degradation.

(9) $$ln\frac{rate\;1}{rate\;2}=-\frac{E_{a}}{R}\left(\frac{1}{T_{1}}-\frac{1}{T_{2}}\right)$$

where $E_{a}$ *R* and *T* are activation energy, universal gas constant (8.314 J K^−1^ mol^−1^) and reaction temperature (K).

## 4. Conclusions

The performance of the Fe(VI) process to degrade HBD has been successfully investigated. According to this research, the reactivity of ferrate performance depends on the pH, and a neutral pH has been observed as the best condition for both bromobenzene and chlorobenzene degradation. As for the effect of the molar ratio, the increase in the molar ratio was found to be linearly related to degradation efficiency, while under some conditions (e.g., a ferrate/chlorobenzene molar ratio of 8/1), chlorobenzene can be degraded entirely in less than 10 min. With respect to temperature, 45 °C was observed as the optimal temperature with an activation energy of 81.6 KJ/mol for bromobenzene degradation and 80.540 KJ/mol for chlorobenzene. From the investigation of the degradation of bromobenzene and chlorobenzene by ferrate(VI), it was found that ferrate(VI) was a promising solution in industrial wastewater treatment.

## Data Availability

The authors declare that the data supporting the findings of this study are available within the article.
